# Supplementary material for: Physicochemical Characterization of an Exopolysaccharide Produced by Lipomyces sp. and Investigation of Rheological and Interfacial Behavior
Source: Gels. 2021 Sep 28;7(4):156. doi: 10.3390/gels7040156 (PMC8544488; doi:10.3390/gels7040156)
Supplement: Supplementary file 1 [file gels-07-00156-s001.zip › gels-1373086-supplementary.pdf]

gels

Article

Physicochemical Characterization of an Exopolysaccharide Produced by *Lipomyces* sp. and Investigation of Rheological and Interfacial Behavior

Wentian Li [1], Yilin Guo [1], Haiming Chen [1,2,*], Wenxue Chen [1], Hailing Zhang [3], Ming Zhang [1], Qiuping Zhong [1] and Weijun Chen [1,4,*]

Supplementary Materials:

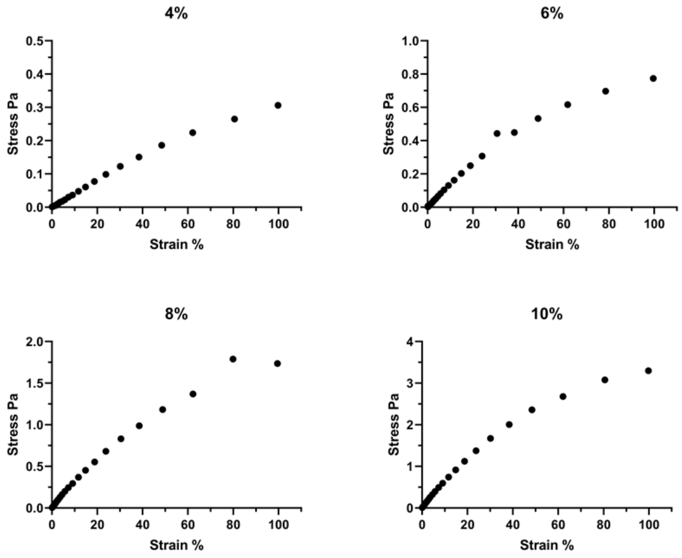

Figure S1. Measurement of linear viscoelastic region.
